# In Response to: Poisonings with Suicidal Intent Aged 0–21 Years Reported to Poison Centers 2003–12

**DOI:** 10.5811/westjem.2015.11.28886

**Published:** 2016-01-21

**Authors:** Daria Falkowitz

**Affiliations:** North Shore University Hospital, Department of Emergency Medicine, Division of Medical Toxicology, Manhasset, New York

To the Editor:

It was with great interest that we read the paper by Sheikh et al. that attempted to use Poison Control Center (PCC) data to explore the clinical features of self-poisonings with suicidal intent in children. Understanding the features of youth-attempted suicide by poisoning is necessary to effectively identify and treat those patients at highest risk for serious outcomes.

The study was able to characterize several clinical features of the 52.2% of subjects in the 15–18 year old age group. We question why the authors performed a subgroup analysis on children <10 years of age, since this group represented <1% of the study patients. It seems that an in-depth analysis of the 15–18 year old age group would have yielded a more useful conclusion. As clinicians, we view the most clinically significant population as those who attempted and ultimately completed suicide. The data provided to readers in this regard were incomplete. Details regarding the exact age, substance ingested and details surrounding the cause of death would be useful in determining risk factors of the population and how the substance may have contributed to mortality. Given the relatively small number of deaths, this information could have easily been provided for a more complete assessment.

Finally, the authors conclude that undiagnosed attention deficit hyperactivity disorder (ADHD) predisposes pediatric patients to self-harming behaviors. This conclusion is completed unsupported by any data. No information about medical history is mentioned and readers cannot assume that patients that overdosed on ADHD medications did so because they had the diagnosis themselves. Medications may be from siblings, friends or any other number of possibilities. Furthermore ADHD medications have a variety of clinical applications outside of treatment for ADHD.

This study was further hindered by the inherent limitations of analyzing PCC data. We suggest that it could have been strengthened by focusing on the group that is at highest risk, providing past medical history and prescribed medications, and focusing on mortality as a more significant outcome. Providing readers with this information would have strengthened the conclusions and enabled further interpretation of the data.

